# Orthodontic treatment protocol versus Peer Assessment Rating: Assessing the quality of orthodontic treatment

**DOI:** 10.1177/14653125241268763

**Published:** 2024-08-16

**Authors:** Jonathan D Shelswell, Brian M Kelly, Trevor M Hodge, Sophy K Barber

**Affiliations:** 1 Department of Orthodontics, University of Leeds, Leeds, UK; 2 NHSBSA Dental Services, Newcastle upon Tyne, UK; 3 Department of Orthodontics, Leeds Teaching Hospitals NHS Trust, Leeds, UK

**Keywords:** epidemiology in orthodontics (including occlusal indices), health services and quality of life aspects, economics of orthodontic delivery systems, risk/benefit assessment

## Abstract

**Objective:**

To apply the Peer Assessment Rating (PAR) to cases that have been assessed by the NHS Business Service Authority (NHSBSA) using the orthodontic treatment protocol (OTO), then compare the NHSBSA outcome assessment with weighted (W) and unweighted (U) PAR scores.

**Design:**

Cross-sectional study.

**Setting:**

UK.

**Cases:**

Anonymised orthodontic cases submitted to the NHSBSA.

**Methods:**

A sample of 30 reports from 2021/2022 were randomly selected to include different standard of treatment grades. The records were de-identified and the pre- and post-treatment study models were PAR scored by a calibrated assessor.

**Results:**

The mean percentage change in PAR was higher in cases from green reports (W: 78%; U: 79%) than amber (W: 68%; U: 67%) and red reports (W: 65%; U: 65%). Alignment and poor buccal segment interdigitation were the most reported concerns for cases included in the red and amber graded reports. A residual increased overjet was the most common occlusal feature leading to PAR scores not being more than 70% improved. Only slight agreement was shown between OTP and PAR using the kappa statistic, and the chi-square statistical test found that outcome measures are statistically significantly different.

**Conclusion:**

There are fundamental differences between OTP and PAR, and general agreement between them has not been demonstrated. The NHSBSA Report provides a more critical outcome assessment than PAR, identifying elements that are not assessed or measured by the PAR index.

## Introduction

In the 1980s, before the widespread consistent use of outcome measures, there were concerns that National Health Service (NHS) orthodontic treatment in the UK was, at times, being undertaken unnecessarily and to a poor standard. The concerns were substantiated after the publication of the Schanschieff Report (Schanschieff, 1986). This led to the development of two occlusal indices: the Index of Orthodontic Treatment Need (IOTN) (Brook and Shaw, 1989) and the Peer Assessment Rating (PAR) index (Richmond et al., 1992a). The PAR index was designed in 1987 by a working group of 10 experienced orthodontists, the British Orthodontic Standards Working Party (Richmond et al., 1992a). This group analysed over 200 pre-, mid- and post-treatment study models to determine which aspects of a malocclusion should be included within the index, originally including 11 components. A score is assigned to each component, weighted and then combined, with a higher score representing a greater deviation from a normal occlusion. Excellent inter- and intra-examiner reliability and validity have been demonstrated when using the index (Richmond et al., 1992a).

Weightings were devised at the time of the development of the PAR index (Table 1). These were derived using multiple regression analysis to most accurately reflect the consensus of the 74 dentists examining the validity of the index, acknowledging that the weightings may be changed in the future as orthodontic opinion and treatment standards change (Richmond et al., 1992a). A lack of predictive power between examiners resulted in no weighting being applied to some occlusal components, such as buccal segment spacing and crowding, effectively eliminating them from the index (Richmond et al., 1992a). There have been suggestions that other weightings are too great, such that the change in PAR score is overly sensitive for any case with an increased overjet (Hamdan and Rock, 1999; Richmond et al., 1992b). Attempts have been made to develop different weightings according to the incisor classification and comparisons between orthodontists in Europe and the United States, but the original weightings are used most commonly in the UK (DeGuzman et al., 1995; Hamdan and Rock, 1999). The index is designed to look at a large group of patients rather than an individual patient’s outcome, as there are a small number of patients where the PAR score does not fully represent the clinical improvement obtained (NHS England, 2015).

**Table 1.** Components of Peer Assessment Rating.

Component	Weighting
Upper anterior segment alignment	X1
Lower anterior segment alignment	X1
Buccal occlusion • Anteroposterior • Transverse • Vertical	X1
Overjet	X6
Overbite	X2
Centreline	X4

It is not practicable for all orthodontic cases to be treated to a perfect occlusion, so there was a need to establish a ‘worthwhile’ improvement in PAR score. Richmond et al. (1992b) determined that a change in PAR score of 22 was ‘greatly improved’; however, some cases that start with a low PAR score can still have a high treatment need and complexity despite never reaching the ‘greatly improved’ threshold on a nomogram; therefore, percentage changes are often described instead. Currently, a PAR score improvement greater than 70% represents a very high standard of treatment, less than 50% shows an overall poor standard of treatment and less than 30% means the patient’s malocclusion has not been improved by orthodontic intervention. It has been suggested that 75% of completed cases should show at least 70% improvement in the PAR score after treatment, with fewer than 3% of cases having less than 30% reduction in PAR (McMullan et al., 2003).

It is a statutory requirement of the NHS orthodontic contract that all performers in England and Wales monitor treatment outcomes using PAR (UK Statutory Instrument, 2005, SI 2005/3361). Each provider must assess 20 cases plus 10% of the remainder of their caseload every year. For contracts commissioned since 2019, the 20 cases are selected by the NHSBSA. For those commissioned in 2006, and not subsequently re-tendered, the cases can be selected by the provider with the recommendation that the first 20 completed cases and every 10th case thereafter are included. PAR is also the primary method used for assessing treatment outcome in secondary care.

The NHS Business Services Authority (NHSBSA) has responsibility for clinical monitoring and reporting (NHSBSA, 2022; UK Statutory Instrument, 2006, SI 2006/596). Trained and calibrated specialist orthodontist assessors evaluate five completed cases per performer on at least one occasion every 3 years, using full records (including pre- and post-treatment study models, radiographs, photographs and FP17DCO form) and an Orthodontic Case Assessment (OCA) form (NHSBSA, 2023). The five cases are selected by the NHSBSA and normally comprise the five most recently reported 21 UOA case completions. Only cases reported as ‘treatment completed’ are selected for assessment. Cases reported as ‘treatment abandoned’ (where the patient decided to terminate treatment) or ‘Treatment discontinued’ (where the performer decided to terminate treatment before the objectives were achieved) are excluded from the sample (NHSBSA, 2024). Performers are also invited to leave comments if they wish for these to be taken into consideration when the records are assessed, such as treatment with limited objectives or poor patient cooperation.

Reports are generated based on three categories: (1) clinical records; (2) treatment need (IOTN); and (3) standard of treatment. The final report provides a grading of red (unsatisfactory, requiring further investigation), amber (satisfactory, but where reservations were expressed) or green (good, satisfying all relevant criteria) for each of the three categories. The NHSBSA orthodontic advisers do not assess the standard of treatment using the PAR index but instead use the British Orthodontic Society and Department of Health (BOS/DH) ‘orthodontic treatment protocol’ (OTP) (Table 2) (Department of Health, 2005). Within the OTP, some ratings are binary and others are more subjective. The OTP was agreed between the Department of Health and representatives of the BOS at the inception of the 2006 NHS orthodontic contract to reflect the body of specialist orthodontic opinion at the time; the OTP was not subjected to validity and discrimination assessment. Grades are not assigned to occlusal traits; instead, assessors more subjectively compare the standard of treatment against the ideal treatment aims defined in the OTP. In the report, a short summary of the successes and shortcomings of treatment is provided for each of the five cases. Example excerpts from these summaries are given in Table 3.

**Table 2.** The aims of orthodontic treatment, as stated in the BOS/DH Orthodontic Treatment Protocol, published in the Commissioning Specialist Dental Services (Revised) Gateway Reference 5865, Appendix 2.

**Orthodontic treatment protocol**
Treatment will normally be completed with fixed orthodontic appliances in both arches.
Treatment of a single arch should only be undertaken where this would be sufficient to achieve the requisite quality of outcome.
Removable orthodontic appliances may be used for minor tooth movements and as an adjunct to fixed appliances.
Functional orthodontic appliances will be used when necessary to correct antero-posterior occlusal discrepancies.
Anchorage reinforcement with lingual arches, palatal arches and extra-oral traction should be used when appropriate.
A high standard of outcome is expected. The following principles indicate the features to be aimed at in treating a case: • The dental arches should be fully aligned with all rotations and mesio-distal angulations corrected. • The occlusal planes should be levelled. • The overjet and overbite should normally be corrected to give cingulum contact between the incisors. • The bucco-lingual or labio-lingual inclination of the teeth should be within the normal range except where dento-alveolar compensation for skeletal discrepancies is necessary. • The centrelines should where practical be coincident. • The buccal segments should interdigitate fully. • Extraction spaces should be closed with roots of adjacent teeth parallel. • Crossbites should normally be corrected. • Centric occlusion should correspond closely with centric relation. • The lower inter-canine width should not be increased. Lower incisors should not be advanced if they are already proclined, and in general should not be advanced more than approximately 2 mm unless there is evidence that they are abnormally retroclined. Expansion beyond these limits should be the exception and only undertaken with informed consent regarding the risk of instability and the likely need for permanent retention.
Retainers should be fitted and supervised as required to maintain tooth position.
Treatment outcome in individual cases will be assessed according to the above principles. It is acknowledged that it is not possible to achieve an ideal occlusion in every case and the PAR index or an alternative index will therefore be used additionally to allow a profile of the practitioner’s overall treatment standards to be developed.

**Table 3.** Example excerpts from NHSBSA red, amber and green reports.

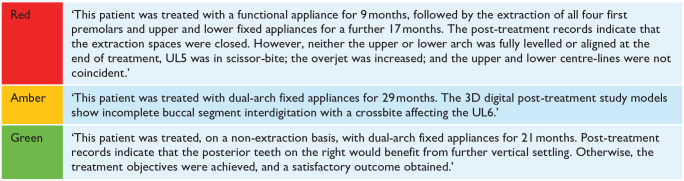

All advisers/assessors participate in training as part of their induction, assessing cases using the criteria outlined by the OTP and subjectively grading each case red, amber or green. There is no specific formula to determine the overall RAG grade; this is done subjectively, based on the findings from each of the five cases and the adviser’s opinion of the fairest overall grade. During their first 3 months of employment, every adviser’s report is reviewed by the senior orthodontic adviser (SOA). Thereafter, reports are reviewed by the SOA at the request of the adviser. There are quarterly peer review and calibration meetings where disputed reports are reviewed and consensus agreed.

A summary of the report is sent to commissioners to facilitate discussion with the provider (contract-holder) if necessary. If concerns are identified, a further five case records are requested for a targeted assessment. These are chosen by the NHSBSA, again being the next five most recently reported 21 UOA completions. Per annum, 350 performers are clinically monitored in this way, on a 3-year rolling programme. For performers with all green grades, monitoring every 36 months is undertaken, whereas performers with amber or red grades in the standard of treatment categories are monitored every 24 and 12 months, respectively.

To date, no comparisons have been made between the two methods currently used for assessing NHS treatment outcomes in the UK. Comparing the scores for treated cases using PAR and OTP will provide information about their ability to assess the quality of orthodontic treatment outcome. Using a valid and discriminative tool for assessing outcome is essential to monitoring and improving the standard of treatment.

The objective of this study was to apply the Peer Assessment Rating (PAR) to cases that had been assessed by the NHSBSA, then compare the NHSBSA standards of treatment outcome assessment with weighted (W) and unweighted (U) PAR scores.

## Cases and methods

### Design

This was a cross-sectional study using de-identified orthodontic cases submitted to the NHSBSA. The University of Leeds Dental Research Ethics Committee confirmed ethical approval was not necessary for this research as it was secondary use of de-identified data.

### Cases

A total of 30 NHSBSA reports, completed by six different NHSBSA advisers, during 2021/2022 were consecutively selected from within each standard of treatment grade, to include 10 green, 10 amber and 10 red reports, representing approximately 10% of the yearly number of reports produced by the NHSBSA. This sample size was determined by the need to select a manageable sample of reports with an equal number of red, amber and green grades. Due to the nature of how the NHSBSA gathers records and produces reports, consecutively sampling according to their RAG grading is effectively random. Each report contained five completed cases as selected by the NHSBSA. Cases reported as abandoned or discontinued were excluded from the NHSBSA sample. The orthodontic administrative team within the NHS Dental Services at NHSBSA de-identified the data and transferred relevant records (pre- and post-treatment digital study models, the OCA form, as well as radiographs and intra-oral photographs where available) to the research team using the Egress Secure Workplace (Egress Software Technologies Ltd., 2023).

Of the 10 reports with a green standard of treatment grade, pre- and post-treatment study models were submitted to the NHSBSA and available to PAR score for all 50 cases. Of the 50 cases in amber reports, complete pre- and post-treatment study models were not submitted to the NHSBSA for two cases. A total of eight pre- and post-treatment study models were incomplete for cases included in the red reports.

### Outcome assessment

**Figure 1.**
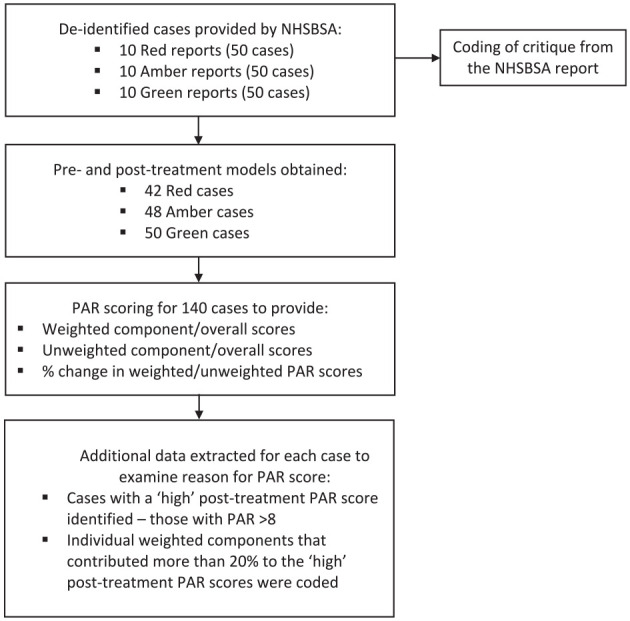
Summary of data collection, extraction and analysis.

For each completed case within the reports, the pre- and post-treatment digital study models were PAR scored by a trained and calibrated assessor (JS), viewing STL files using MeshLab open source software (MeshLab, 2024). PAR scoring of digital study models has been shown to be valid and reliable (Mayers et al., 2005). Overall, the PAR index could be applied to 140 (93.3%) out of a possible 150 pairs of pre- and post-treatment study models, due to incomplete records being submitted to the NHSBSA (Figure 1). The PAR scores for each component of PAR, the overall weighted and unweighted scores, and the percentage changes in the pre- and post-treatment weighted and unweighted PAR scores were recorded in an Excel spreadsheet using a de-identifying case number. Alongside this, the critical summary of that specific case was taken directly from the NHSBSA report.

The mean percentage changes in weighted and unweighted PAR scores were calculated for each report (five cases) to provide overall percentage changes for the cases included in red, amber and green reports, respectively. Separate one-way ANOVAs were also performed to compare percentage changes in weighted and unweighted PAR scores for cases in red, amber and green reports. The effect of specific occlusal components on the reduction in PAR scores and high post-treatment PAR scores were recorded.

### Coding

From the critical summary in the NHSBSA report, specific shortcomings in treatment outcome were identified and coded, such as ‘poor buccal segment interdigitation’ or ‘residual increased overjet’. The frequency of specific criticisms in the NHSBSA reports was recorded for all cases.

### Constraints

Components were only coded if they contributed more than 20% to the reduction in the weighted PAR scores, or if they made up more than 20% of a high post-treatment weighted PAR score. Post-treatment PAR scores were considered ‘high’ if they were greater than 8. These arbitrary limits were chosen after discussion within the research team for two main reasons: first, to avoid analysis of small occlusal changes, which contributed insignificantly (deemed as ⩽20%) to the overall change in PAR score, therefore limiting ‘noise’ during data analysis; and second, to prevent unnecessary analysis of acceptable post-treatment PAR scores (deemed as ⩽8). As an example, if the post-treatment PAR score was 10, individual post-treatment weighted components would need to score 3+ to be coded. Since the combined anteroposterior buccal occlusion very commonly scores at least 2 overall, even in well-finished cases, using a ‘high’ post-treatment PAR score cut-off below 8 would over-analyse this occlusal component.

### Agreement

The NHSBSA assessors are experienced specialist orthodontists, trained and calibrated in the use of the OTP and the PAR index. No further reliability testing was undertaken for the grading of the records using the OTP. Two months after the original data collection, the first 20% of digital study models to be originally PAR scored were reassessed by the same assessor (JS). A Bland–Altman plot was used to determine the intra-rater reliability.

## Results

### Reliability

**Figure 2.**
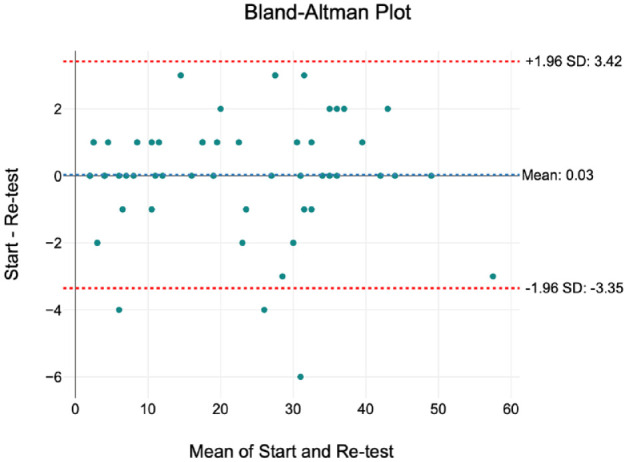
Bland–Altman plot to determine intra-rater agreement.

The Bland–Altman plot demonstrated a good level of intra-rater agreement, with a small bias of 0.03 (Figure 2).

### Baseline characteristics

The mean pre-treatment weighted and unweighted PAR scores for each standard of treatment grade are presented in Table 4. A one-way ANOVA revealed a statistical difference in weighted (F(2, 137) = [4.67], *P* = 0.011) and unweighted (F(2, 137) = [3.25], *P* = 0.042) pre-treatment PAR scores. Tukey’s HSD test for multiple comparisons found significant differences in weighted pre-treatment PAR scores between red and amber reports (*P* = 0.015) and red and green reports (*P* = 0.027). Significant differences were also found between unweighted pre-treatment PAR scores for cases in red and green reports (*P* = 0.045).

**Table 4.** Comparison between changes in PAR scores for cases, grouped by OTP category.

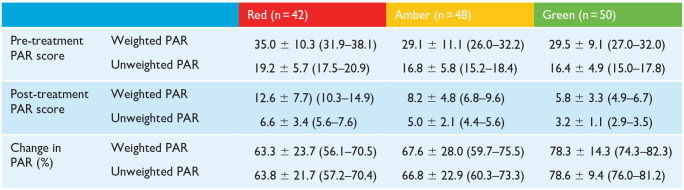

Values are given as mean ± SD (95% CI).

CI, confidence interval; OTP, orthodontic treatment protocol; PAR, Peer Assessment Rating.

### Changes in PAR score

The mean post-treatment PAR scores for each standard of treatment grade are also presented in Table 4. A one-way ANOVA showed a statistical difference between post-treatment weighted PAR scores (F(2, 137) = [17.94], *P* < 0.001), with Tukey’s HSD test for multiple comparisons showing significant differences between cases in red and amber reports (*P* < 0.001), and between red and green reports (*P* < 0.001). No significant differences were shown in post-treatment weighted PAR scores between amber and green reports (*P* = 0.091).

When unweighted post-treatment PAR scores were compared between the standard of treatment grades, a one-way ANOVA again reported a statistical difference (F(2, 137) = [23.80], *P* = 0.000). Tukey’s HSD test found significant differences between cases in red and amber reports (*P* = 0.002), red and green reports (*P* < 0.001) and amber and green reports (*P* = 0.002).

The mean percentage change in weighted and unweighted PAR scores was greater for green cases than amber or red (Table 4). A one-way ANOVA revealed that there was a statistical difference in percentage change in weighted PAR scores between at least two of the three standard of treatment gradings of reports (F(2, 137) = [5.48], *P* = 0.005). Descriptive data supporting the ANOVA is represented in Table 5. Tukey’s HSD test found that the mean percentage change in weighted PAR score was significantly different (*P* = 0.005) between cases in red reports (63.3% ± 23.7%, 95% CI = 56.1–70.5) and green reports (78.3 ± 14.3, 95% CI = 74.3–82.3). No statistical differences (*P* = 0.622) were found in the mean percentage change in weighted PAR scores between red and amber (67.6 ± 28.0, 95% CI = 59.7–75.5) reports, or amber and green reports (*P* = 0.064).

**Table 5.** Descriptive statistics of the percentage change in weighted and unweighted PAR scores for the three standard of treatment grades.

	N	Mean ± SD	95% CI for mean	Minimum (%)	Maximum (%)
*Change in weighted PAR scores (%)*					
Green	50	78.3 ± 14.3	74.3–82.3	33	96
Amber	48	67.6 ± 28.0	59.7–75.5	−75	95
Red	42	63.3 ± 23.7	56.1–70.5	−21	96
*Change in unweighted PAR scores (%)*					
Green	50	78.6 ± 9.4	76.0–81.2	50	93
Amber	48	66.8 ± 22.9	60.3–73.3	−57	90
Red	42	63.8 ± 21.7	57.2–70.4	−9	92

CI, confidence interval; OTP, orthodontic treatment protocol; PAR, Peer Assessment Rating.

A further one-way ANOVA compared the percentage change in unweighted PAR scores. This also found a statistical difference between at least two of the three categories of reports (F(2, 137) = [8.23], *P* < 0.001). Tukey’s HSD test found that the mean percentage change in unweighted PAR score was significantly different (*P* = 0.001) between cases in red (63.8 ± 21.7, 95% CI = 57.2–70.4) and green (78.6 ± 9.4, 95% CI = 76.0-81.2) reports and amber (66.8 ± 22.9, 95% CI = 60.3–73.3) and green reports (*P* = 0.009). No statistically significant differences (*P* = 0.711) were found for the percentage change in unweighted PAR scores between red and amber reports.

### Agreement of OTP and PAR

Cases included in green reports were more consistently treated to a high standard (>70% improvement in PAR score) (Table 6). Substantially more cases were treated to a poor standard (30%–50% improvement in PAR) in red reports (24%), than amber (4%) or green (4%) reports. All cases in green reports saw an improvement in the post-treatment PAR score of at least 30%, whereas 6% of amber report cases and 5% of red report cases did not show an improvement in the malocclusion.

**Table 6.** Comparison between standards of treatment according to change in weighted PAR scores, grouped by OTP category.

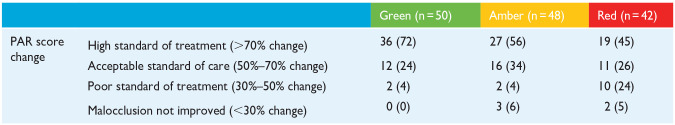

Values are given as n (%).

OTP, orthodontic treatment protocol; PAR, Peer Assessment Rating.

The degree of agreement between OTP and PAR can also be quantified using kappa, using the assumptions that a green report corresponds to a high standard of treatment, an amber report corresponds to an acceptable standard of treatment, and a red report corresponds to a standard of treatment that is poor or not improved. This is not strictly comparable, given a subjective decision is made by the NHSBSA advisers about the quality of treatment of all five cases when deciding on the standard of treatment grade. Nonetheless, applying this comparison produces a kappa value of 0.17, indicating only slight agreement between OTP and PAR. Similarly, using the same assumptions, the chi-square statistical test can be applied, providing a statistic of 14.37 with a *P* value of 0.006. This suggests that the findings of the OTP and PAR are statistically significantly different.

### Component impact

**Figure 3.**
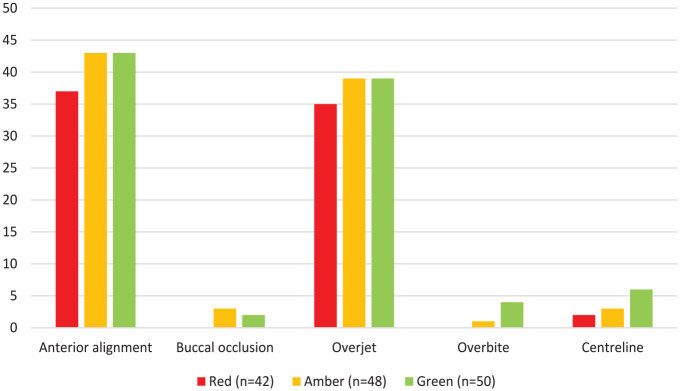
The frequency that components of the PAR index contribute to a greater than 20% reduction in PAR score. PAR, Peer Assessment Rating.

Figure 3 summarises how the individual occlusal components of the PAR index contribute to the reduction in the PAR score. For cases in red, amber and green reports, the upper and lower anterior segment alignment most frequently contribute to a reduction in the PAR score of at least 20%. This is closely followed by overjet. The buccal occlusion, overbite and centrelines all contribute substantially less to a reduction in PAR scores for all cases. Of the five cases where improvements to the buccal occlusion contributed more than 20% to the reduction in PAR score, when the elements of buccal occlusion were considered separately, only improvements to the transverse occlusion individually contributed more than 20%.

**Figure 4.**
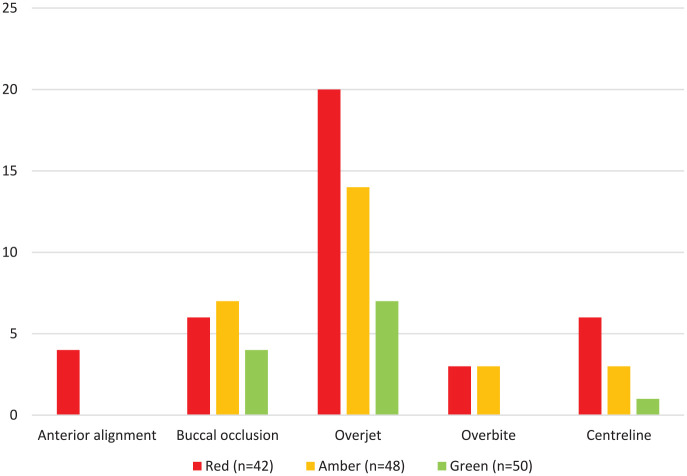
The frequency that components of the PAR index contributed to a high post-treatment PAR score (greater than 8).

Within the 42 pairs of study models in the red reports, there were no examples where improvements in the buccal occlusion or overbite contributed to a greater than 20% reduction in the PAR score. The most common components contributing greater than 20% to a high post-treatment PAR score (deemed to be greater than 8) was a residual increased overjet followed by buccal occlusion for cases in all categories (Figure 4). Out of the 17 cases where the overall buccal occlusion contributed greater than 20% to a high post-treatment PAR score, the antero-posterior buccal occlusion was the cause in nine cases, the transverse buccal occlusion was the cause in three cases and the vertical buccal occlusion did not contribute. Anterior alignment, overbite and centrelines contribute less often to a high post-treatment PAR score.

Many of the comments in the critical summaries provided in the NHSBSA reports were similar between cases in red and amber reports. The comments were grouped in one of three themes: occlusal outcome; treatment method; and records submitted to the NHSBSA (Table 7). The most frequent occlusal outcome concern in all reports was poor buccal segment interdigitation, followed by alignment for red and amber reports only. A residual increased overjet was criticised considerably more often in cases in red reports (57%) than in amber reports (13%). Of the cases identified as having a residual increased overjet in the NHSBSA reports (two green, six amber and 24 red), both green cases, all six amber cases, and 17/24 red cases were also critiqued for poor buccal segment interdigitation. The choice of extraction was not questioned in any cases in amber or green reports but was mentioned for 7 (17%) cases in the red reports.

**Table 7.** Frequency of concerns raised by the NHSBSA in reports where the standard of treatment was graded as red or amber.

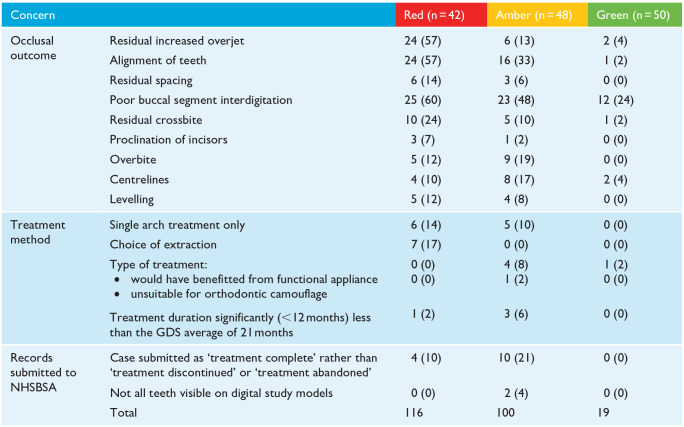

Values are given as n (%).

GDS, General Dental Service.

The third most frequently raised concern (in 21% of cases) in amber reports was that performers had indicated treatment had been successfully completed, when the comments provided by the performer suggested that the treatment had been abandoned or discontinued.

## Discussion

### Summary

This study found poorer improvements in mean PAR scores for cases included in NHSBSA reports where the standard of treatment was graded as unsatisfactory. A much higher proportion of cases in red reports showed only a 30%–50% improvement in PAR score, indicating agreement between the OTP and PAR index. Furthermore, although the overall number of treated cases showing the poorest (<30%) improvements in PAR score was low (n = 5, 3.6%), all were from reports with a red or amber score for standard of treatment.

There was less agreement when the individual components of PAR scores were compared with the critical summaries provided in the NHSBSA reports. For example, in 35/42 cases in red reports, a reduction in the overjet led to a greater than 20% improvement in PAR score. However, for the same reports, applying the OTP led to 24 cases being criticised for having a residual increased overjet. For cases in red and amber reports, poor buccal segment interdigitation was the most frequent post-treatment criticism, discussed in 48/90 (53%) cases. For the same 90 cases, the buccal occlusion contributed to more than 20% of the post-treatment PAR score in only 18 (20%) cases. Reducing a greatly increased overjet (OJ), even to an OJ that is still greater than average, is likely to contribute more than 20% to the improvement in PAR but may still be critiqued in an NHSBSA report. The association between a residual increased overjet and poorer buccal segment relationship is also a logical finding.

### Limitations

There are several limitations that should be acknowledged in this study. Although a high level of intra-rater reliability was demonstrated, data collection was only undertaken by one non-blinded assessor, which is a potential source of observer and measurement bias. Similarly, bias may have been introduced by reliability testing the first 20% of cases, rather than re-scoring a random sample from all cases.

An arbitrary contribution of 20% was chosen to determine which specific occlusal features contributed sufficiently to a reduction in, or high post-treatment, PAR score. Similarly, the research team determined that a residual post-treatment PAR score of at least 8 was ‘high’. This was advantageous because it prevented analysis of insignificant occlusal changes and minor components of acceptable post-treatment PAR scores. However, it may have introduced a selection bias in favouring the more heavily weighted PAR components.

Although kappa and chi-square statistical tests were undertaken and provided further context when comparing OTP and PAR, these can only be undertaken using certain assumptions, such as an NHSBSA amber standard of treatment grade most closely correlates with an ‘acceptable’ change in PAR score of 50%–70%. Although this seems logical, it is not necessarily factual. Since the standard of treatment grade relates to the five individual cases included within that report, it is quite possible that four of the five cases were treated to a high standard according to the change in PAR score, but the fifth case led to an overall amber standard of treatment grade. Finally, some of the treatment method concerns raised in the NHSBSA comments, particularly ‘choice of extraction’ and ‘would have benefitted from functional appliance’, could be deemed to be assessor dependent and highly subjective even within the experienced, trained and calibrated NHSBSA assessor team.

### Implications for clinical practice

The PAR index has several advantages explaining its widespread use as a tool in audit and research. It has been shown to have excellent inter- and intra-rater reliability and can also be applied by non-clinicians (Richmond et al., 1992a). However, the index only assesses the position of teeth on study models, and other factors are important when determining the success of treatment, including aesthetics, the inclination of anterior teeth, decalcification and gingival recession (Otuyemi and Jones, 1995a, 1995b). There are several aspects of the OTP that cannot be measured from study models alone, including:

- Normal inclination except where dento-alveolar compensation for skeletal discrepancies is necessary.
- Extraction spaces closed with roots of adjacent teeth parallel.
- Lower incisors should not be advanced more than approximately 2 mm unless there is evidence that they are abnormally retroclined.

Overall, the results highlight that there are fundamental differences between the two occlusal outcome measures, and both have merits and limitations. Although the PAR index is an objective numerical outcome measure, the OTP arguably gives a more comprehensive and nuanced opinion about the success of treatment. However, a possible flaw of the OTP is its subjectivity and lack of accessibility to the orthodontic workforce, requiring a greater degree of assessor training and calibration. There is also an argument that the OTP should be reviewed and updated, particularly in view of modern trends towards changes in arch form and permanent retention, but it is conceivable that the discretion of NHSBSA assessors provides an element of fairness, particularly if mitigating comments provided by performers are considered.

### Implications for research

The PAR index does not adequately assess incisor inclination, extraction space closure or buccal segment alignment (Hinman, 1995). Although buccal occlusion is measured in the PAR index, the results of this study suggest it is not sufficiently discriminative to record when the post-treatment buccal occlusion is poor. The possible benefits of revision of the weightings of the PAR index have been discussed (Hamdan and Rock, 1999) and increasing the weightings of the buccal segment alignment and occlusion would help more closely correlate PAR scores with current practice and expected standards. The results of this study found the overall average percentage change in PAR scores was similar regardless of whether components were weighted or unweighted. This may indicate that neither unweighted PAR nor the current PAR weightings are appropriate, and research into a new re-weighting of PAR would be most beneficial.

Other established occlusal outcome measures may also have a better agreement with the OTP, and this should be investigated further. This would most notably include the Index of Complexity, Outcome and Need (ICON), which was developed to be a valid single index that could assess both treatment need and outcomes (Daniels and Richmond, 2000). The components and weightings of ICON address some of the deficiencies of PAR, including buccal segment anteroposterior occlusion and the scoring of residual extraction spacing. Nevertheless, these potential advantages have been apparent since the inception of ICON, but there has been no widespread uptake in its use in the UK, with IOTN and PAR being the most popular occlusal indices. Indeed, there is a regulatory requirement for them to be applied for NHS orthodontic cases (UK Statutory Instrument, 2005, SI 2005/3361). A revised weighting of PAR may be a more pragmatic approach to manage the current discrepancy between outcome measures, and one that would be more readily accepted within UK orthodontic practice.

## Conclusion

There are fundamental differences between OTP and PAR, and general agreement between them has not been demonstrated. The PAR index is an objective numerical outcome measure, whereas the NHSBSA reports, with their use of the OTP, provide a more critical outcome assessment, identifying elements that are not assessed or measured by the PAR index.
